# “This behavior strikes us as ideal”: assessment and anticipations of Huisman (2022)

**DOI:** 10.3758/s13423-023-02299-x

**Published:** 2023-08-01

**Authors:** Alexandra Sarafoglou, František Bartoš, Angelika Stefan, Julia M. Haaf, Eric-Jan Wagenmakers

**Affiliations:** 1 Department of Psychology, University of Amsterdam, Nieuwe Achtergracht 129B, 1001 NK Amsterdam, Netherlands; 2 Department of Psychology, Universität der Bundeswehr München, Munich, Germany

**Keywords:** Bayes factors, P-values, Hypothesis testing, Evidential strength, Jeffreys-Lindley paradox

## Abstract

Huisman (*Psychonomic Bulletin & Review*, 1–10. 2022) argued that a valid measure of evidence should indicate more support in favor of a true alternative hypothesis when sample size is large than when it is small. Bayes factors may violate this pattern and hence Huisman concluded that Bayes factors are invalid as a measure of evidence. In this brief comment we call attention to the following: (1) Huisman’s purported anomaly is in fact dictated by probability theory; (2) Huisman’s anomaly has been discussed and explained in the statistical literature since 1939; the anomaly was also highlighted in the *Psychonomic Bulletin & Review* article by Rouder et al. (2009), who interpreted the anomaly as “ideal”: an interpretation diametrically opposed to that of Huisman. We conclude that when intuition clashes with probability theory, chances are that it is intuition that needs schooling.

The article by Huisman (2022) centers on an important question: “Are *p*-values and Bayes factors valid measures of evidential strength?”. Huisman first introduces two criteria for a measure of evidential strength, the critical one being that when the alternative hypothesis $\mathcal {H}_1$ is true, the expected evidence in its favor should increase monotonically with sample size. Huisman then demonstrates that Bayes factors violate this criterion: when the true effect size is small but non-zero, an increase in sample size will initially be taken to support the incorrect model $\mathcal {H}_0$; only when sample size increases still further will the data gradually overcome this dip in evidence and indicate support for the correct model $\mathcal {H}_1$ (see Fig. 1). Huisman concludes that the Bayes factor is not a valid measure of evidence. This is a bold claim that flatly contradicts over 80 years of work in the statistical literature, some of which was presented in *Psychonomic Bulletin & Review* (e.g., Myung and Pitt, 1997; Rouder et al., 2009; Vandekerckhove et al., 2018; Wagenmakers et al., 2018).

**Fig. 1:**
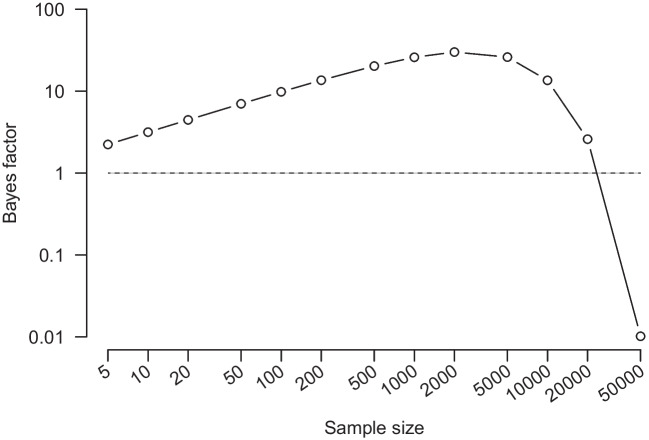
Reproduction of Figure 5 from Rouder et al. (2009). BIC-approximation Bayes factor BF for a one-sample t-test of a true small sample size, d = 0.02, with increasing sample size

It is true that the non-monotonicity identified by Huisman may initially strike researchers as surprising. However, Huisman is far from the first to highlight this pattern, and the purported anomaly has been discussed and explained in the literature since 1939 (Jeffreys, 1939); notably, earlier work on the anomaly deemed it “ideal” (Rouder et al., 2009). Here we (a) discuss the meaning of evidential strength; (b) show that Bayes factors follow from basic probability theory; (c) provide intuitions for the presence of the purported anomaly; (d) cite a subset of the relevant literature that already presented the anomaly and interpreted it in a favorable light.

## Bayes factors and the meaning of evidence

In the introduction, Huisman (2022) elaborates on the meaning of evidence:

“Evidential strength is not a very well defined concept. Intuitively, it is the extent by which the collected data can change our opinion regarding the plausibility of a hypothesis of interest; that is, the extent to which, upon the acquisition of that evidence, the hypothesis becomes more plausible or less plausible, or maybe just less implausible or a bit more plausible. Strong evidence can have a large effect on how plausible or implausible we finally judge the hypothesis to be, while weak evidence has little effect.” (p. 1)

Strikingly, this is a word-for-word description of the odds version of Bayes’ rule,

1 $$\begin{aligned} \underbrace{\frac{p(\mathcal {H}_0 \mid \text {data})}{p(\mathcal {H}_1 \mid \text {data})}}_{\begin{array}{c} \text {Posterior odds} \end{array}} = \underbrace{ \frac{p(\text {data} \mid \mathcal {H}_0)}{p(\text {data} \mid \mathcal {H}_1)}}_{\begin{array}{c} \text {Bayes factor } \text {BF}_{01} \end{array}} \,\, \times \,\, \underbrace{ \frac{p(\mathcal {H}_0)}{p(\mathcal {H}_1)}}_{\begin{array}{c} \text {Prior odds} \end{array}}, \end{aligned}$$

in which the change from prior to posterior odds for two rival hypotheses is governed by the *Bayes factor*, a measure of relative predictive performance (Kass and Raftery, 1995; Myung and Pitt, 1997; Wagenmakers et al., 2018). Thus, at first glance, the Bayes factor seems to be an apt and perfectly well defined mathematical representation of Huisman’s intuition of evidence. The conclusion that the Bayes factor is a suitable measure for evidence is echoed in the philosophy of science literature (see also, van Dongen et al., 2022). In fact, the Bayes factor was initially developed with the express purpose of being a valid measure of evidence (e.g., Jeffreys, 1939; Ly et al., 1995) stemming from Jeffreys’s convictions that (1) inference needs to be inductive, (2) a formalisation of induction requires inference to follow probability calculus, and (3) testing a general law requires it be given prior probability. More generally, Evans (2015) argued that evidence is defined as that which ought to cause beliefs to change, and that evidence should therefore be quantified by how much the data mandate a change from prior to posterior beliefs. It therefore appears that a closer inspection is warranted before we deem Bayes factors to be evidentially *invalid*.

## Bayes factors as an exercise in probability theory

In our first example, we demonstrate that a counterintuitive result is not necessarily an invalid result - but that probability theory does not always agree with our intuition. Imagine you are a taking a course on probability theory and encounter the following question on the final exam:

“Bellamy tosses a fair coin. If it lands heads, Bellamy will have the program R generate *n* observations from a binomial process $\mathcal {H}_0$ with chance parameter $\theta = \frac{1}{2}$. If the coin lands tails, Bellamy will first have R draw a random value for $\theta$ from a uniform distribution ranging from 0 to 1; this random value will then be used to generate *n* observations from a binomial process $\mathcal {H}_1$. Based only on the synthetic data (i.e., the number of successes *s* generated from *n* attempts) indicate the change from prior to posterior odds in favor of $\mathcal {H}_0$ over $\mathcal {H}_1$ for the following two scenarios: (a) $s=3$, $n=5$; (b) $s=18$, $n=30$.”

**Fig. 2:**
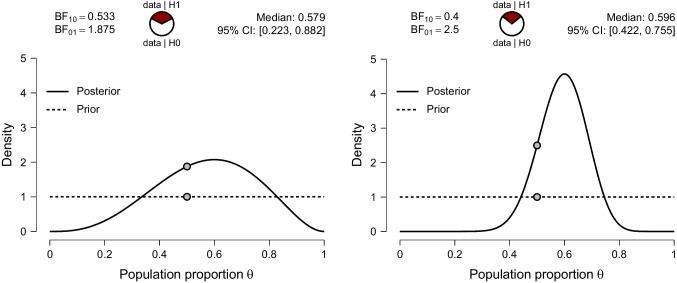
Bayesian inference for Bellamy’s synthetic data comprised of $s=3$ successes out of $n=5$ attempts (left) and $s=18$ successes out of $n=30$ (right). Shown are the prior and posterior distribution for $\theta$ under $\mathcal {H}_1$. The ratio between prior and posterior ordinate at $\mathcal {H}_0: \theta = \frac{1}{2}$ is known as the Savage-Dickey density ratio, which equals the Bayes factor (e.g., Wagenmakers et al., 2010). See text for details. Figures from JASP

This question admits only a single correct answer, and it is given by the Bayes factor – the result of a mechanical application of the law of conditional probability and the law of total probability. For scenario *a*, the inference is summarized by left panel of Fig. 2. The Bayes factor in favor of $\mathcal {H}_0$ is 1.875, indicating that the data $s=3$, $n=5$ are 1.875 times more likely to occur under $\mathcal {H}_0$ than under $\mathcal {H}_1$.

For scenario *b*, the inference is summarized by right panel Fig. 2. The Bayes factor in favor of $\mathcal {H}_0$ is 2.5, indicating that the data $s=18$, $n=30$ are 2.5 times more likely to occur under $\mathcal {H}_0$ than under $\mathcal {H}_1$. These are the correct answers to the exam question.

**Fig. 3:**
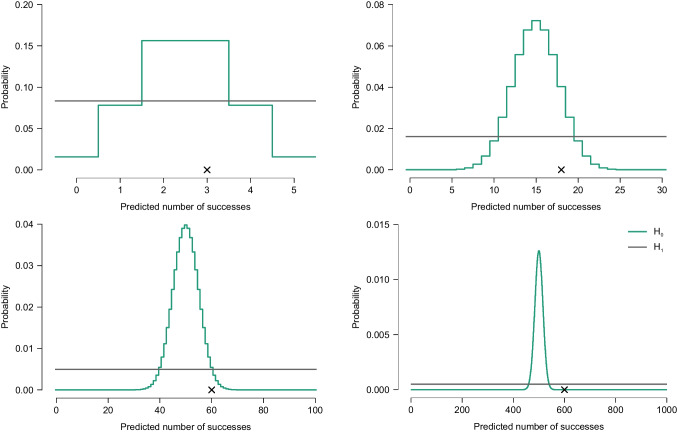
Prior predictive distribution for Bellamy’s synthetic data comprised of $n=5$ attempts (top left), $n=30$ attempts (top right), $n=100$ attempts (bottom left) and $n=1000$ attempts (bottom right). Shown are the prior predictive distributions for the number of success under $\mathcal {H}_0$ and $\mathcal {H}_1$ and an observed proportion corresponding to exactly 60% successes. Figures from JASP

However, suppose that the synthetic data were generated, first by Bellamy’s coin landing tails, and then by drawing $\theta =.60$. Hence, the true data generating process is $\mathcal {H}_1: \theta =.60$. Moreover, in scenarios *a* and *b* we have that $s/n =.60$, such that the sample proportion perfectly reflects the population proportion. However, both scenarios yield evidence in favor of the incorrect model $\mathcal {H}_0$. Also, the larger sample (i.e., scenario *b*) yields *more* evidence for the incorrect model than the smaller sample (i.e., scenario *a*). Figure 3 shows the predicted number of successes for an increasing number of attempts. Only in one of the four scenarios with $n = 1000$ (i.e., bottom right panel) does $\mathcal {H}_1$ predict the number of successes better than $\mathcal {H}_0$. This illustrates the purported anomaly identified by Huisman. However, the fact that this pattern has been labeled counterintuitive, undesirable, anomalous, or invalid takes nothing away from the fact that it is the uniquely correct answer on the exam. Probability theory therefore seems to behave in a manner that conflicts with Huisman’s intuition. In the next section we will try to explain why the anomalous pattern is in fact both desirable and intuitive.

## An intuition for the purported anomaly

The critical pattern described by Huisman can be explained in several ways, some of which are presented in the section that briefly outlines prior work on this topic. Here we wish to emphasize the fact that, in the scenario outlined above, the simple model $\mathcal {H}_0$ is supported by the data because it provides predictions that are more precise than those from the more complex model $\mathcal {H}_1$. By specifying a uniform distribution on $\theta$ that ranges from 0 to 1, $\mathcal {H}_1$ is effectively hedging its bets. When the data undercut extreme outcomes, this therefore harms $\mathcal {H}_1$ more than it does $\mathcal {H}_0$.

Consider a conceptual example loosely based on the one presented in (van Ravenzwaaij and Wagenmakers, 2022). This example demonstrates why, if the alternative hypothesis is true, the null hypothesis initially receives more support when a larger sample is drawn. Guided into a dimly-lit room, you are asked whether or not it contains a living animal (i.e., one animal or no animals). Let $\mathcal {H}_0$ be the hypothesis that the room does not contain any animals; it holds that all movement you observe is either hallucination or due to non-animal causes. Let $\mathcal {H}_1$ be the hypothesis that the room does contain at least one animal; however, in order to make a prediction about the data, $\mathcal {H}_1$ needs to be more specific: what animal are we talking about? Lacking specific information about the animal, $\mathcal {H}_1$ needs to divide its prior probability across several classes of animals. For instance, one may entertain a uniform distribution and assume that large animals, medium-sized animals, small animals, and tiny animals are equally likely *a priori*.

You step into the room and observe...a hint of a movement? It is difficult to tell, but you certainly do *not* see an elephant, a bull, or a sheep. This impression is in line with $\mathcal {H}_0$, but it falsifies part or $\mathcal {H}_1$ (i.e., the part that predicted the presence of large or medium-sized animals). Thus, we have evidence in favor of $\mathcal {H}_0$, as this model did not waste any predictive mass on elephants, bulls, and sheep. We then inspect the room more closely, and find that even rats and mice are nowhere to be found. Again, this impression is in line with $\mathcal {H}_0$, but falsifies part of $\mathcal {H}_1$ (i.e., the part that predicted the presence of small animals). Consequently, the evidence in favor of $\mathcal {H}_0$ increases still further, as this model did not waste any predictive mass on rodents. You then scrutinize the room much more closely, and ultimately you detect a beetle. This observation turns the tables and constitutes decisive evidence in favor of $\mathcal {H}_1$.

There is nothing anomalous, undesirable, or counterintuitive about the above sequence of events. If the true effect is minuscule, but you expect it may be fairly large, you will initially be mislead: $\mathcal {H}_1$ is punished for predicting extreme outcomes that have not materialized. Such is the price of vagueness. In Huisman’s t-test example, these extreme predictions stem from the prior distribution assigned to the effect size parameter. Although the prior distribution is centered on zero, its spread introduces variability to the predictions, assigning density even to high effect size values. As sample size increases, more and more outcomes predicted by $\mathcal {H}_1$ are undercut (i.e., the large predicted effect sizes were not observed), providing even more evidence for $\mathcal {H}_0$. Eventually, the sample size is sufficiently high that the minuscule effect can be differentiated from $\mathcal {H}_0$, which is when the data will start supporting $\mathcal {H}_1$.

With the benefit of hindsight, you may bemoan the fact that you did not assign most prior mass to minuscule effect sizes. “Why on earth did I expect a bull?,” you may wonder, “of course it was going to be a bug. Had I realized this in advance, I would not have expressed so much confidence in $\mathcal {H}_0$ after the first two inspections.” The core of the problem here is not the Bayes factor, but rather the fact that the prior distribution assigned mass to a host of overly optimistic values that the data undercut (van Ravenzwaaij and Wagenmakers, 2022, p. 456). The remedy for this problem is to specify a more informative alternative model. When researchers expect small effect sizes a-priori, they may assign a higher prior density to smaller effect size values, for instance, by more tightly constraining the variance parameter of the effect size prior distribution (in Huisman’s manuscript this parameter is denoted as $\tau ^{2}$ ). In this way, the alternative model becomes more parsimonious and -although the accumulation of evidence in these cases may still be not monotonic- evidence for the true alternative accumulates more quickly. Note that if the variance is maximally constrained, that is, a fixed effect size is assumed under $\mathcal {H}_1$ (e.g., in a power calculation), both models are equally parsimonious and the non-monotonic pattern disappears. Importantly, the anomaly occurs not only in situations were the alternative is specified poorly; the anomaly is a function of having two models where one is more parsimonious than the other.

## Huisman’s anomaly has been discussed by Bayesians for over 80 years

We do not wish to provide an exhaustive review of the articles that have discussed the pattern identified by Huisman (e.g., Morey, 2015; Wagenmakers et al., 2018, p. 52; Wagenmakers and Ly, 2022 and references therein; van Ravenzwaaij and Wagenmakers, 2022, pp. 456–457). Instead we focus on two influential sources: the material presented in Sir Harold Jeffreys’s monograph *Theory of Probability* (1939, 1948, 1961) and the discussion from Rouder et al. (2009) in *Psychonomic Bulletin & Review*. The former introduced Bayes factors to the field of statistics, and the latter introduced Bayes factor *t*-tests to the field of psychology (see also Myung and Pitt 1997). This is not meant to present an argument from authority; rather, it serves to illustrate that the pattern identified by Huisman has been discussed prominently in the literature before, and that its interpretation has been diametrically opposed to that of Huisman: instead of anomalous, the pattern is viewed as desirable and an inevitable consequence of probability theory. Although the term “nonmonotonicity” is not explicitly mentioned in the following quotations, they nevertheless refer to the lack of monotonic behavior of the Bayes factor-the main criticism of Huisman. The excerpts describe that when the true effect size is small, the Bayes factor initially supports neither hypothesis at a sample size of zero, supports the null hypothesis for small samples, and supports the alternative for larger samples, thus implying the absence of monotonicity (see also Fig. 1).

### Discussion in the *Theory of Probability* (1939, 1948, 1961)

Sir Harold Jeffreys pioneered the Bayes factor hypothesis test in the second half of the 1930s (Etz and Wagenmakers, 2017; Wagenmakers and Ly, 2022). His work on Bayesian estimation and testing is presented in *Theory of Probability* (first ed. 1939, second ed. 1948, third ed. 1961). In the 1939 edition, Jeffreys introduces the rationale of the Bayes factor hypothesis test as follows:

“The difficulty pointed out before about the uniform assessment of the prior probability was that even if $\alpha$ [SSBW: the true value of the test-relevant parameter] was 0, *a* [SSBW: the sample estimate] would usually be different from 0, on account of random error, and to adopt *a* as the estimate would be to reject the hypothesis $\alpha = 0$ even if it was true. We now see how to escape from this dilemma. Small values of *a* up to some multiple of *s* [SSBW: the standard error] will be taken to support the hypothesis $\alpha = 0$, since they would be quite likely to arise on that hypothesis, but larger values support the need to introduce $\alpha$. In suitable cases high probabilities may be obtained for either hypothesis. The possibility of getting actual support for the null hypothesis from the observations really comes from the fact that the value of $\alpha$ indicated by it is unique. $\sim q$ [SSBW: $\mathcal {H}_1$] indicates only a range of possible values, and if we select the one that happens to fit the observations best we must allow for the fact that it is a selected value. If *a* is less than *s*, this is what we would expect on the hypothesis that $\alpha$ is 0, but if $\alpha$ could be anywhere in a range *m* it requires that an event with a probability 2*s*/*m* shall have come off. If *a* is much larger than *s*, however, *a* would be a very unlikely value to occur if *a* was 0, but no more likely than any other if $\alpha$ was not 0. In each case we adopt the less remarkable coincidence.” (Jeffreys, 1939, pp. 194–195; annotations based on van Ravenzwaaij and Wagenmakers, 2022; echoed in Jeffreys, 1948, pp. 222, and Jeffreys, 1961, p. 248)

In a later chapter Jeffreys explains that for small effects, initial support for $\mathcal {H}_0$ will turn into support for $\mathcal {H}_1$ as sample size grows large:

“It is worth while to devote some attention to considering *how* a law [SSBW: $\mathcal {H}_0$], once well supported, can be wrong. A new parameter rejected by a significance test [SSBW: Jeffreys’s Bayes factor hypothesis test] need not in fact be zero. All that we say is that on the data there is a high probability that it is. But it is perfectly possible that it is not zero but too small to have been detected with the accuracy yet attained. We have seen how such small deviations from a law may be detected by a large sample when they would appear to have been denied by any sub-sample less than a certain size [SSBW: this refers to the concrete example on p. 269], and that this is not a contradiction of our general rules.” (Jeffreys, 1939, pp. 297–298; annotations based on van Ravenzwaaij and Wagenmakers, 2022; echoed in Jeffreys, 1948, p. 339, and Jeffreys, 1961, p. 367)

### Discussion in *Psychonomic Bulletin & Review* (2009)

Rouder et al. (2009) provide an accessible introduction to Bayes factor hypothesis testing. They present the Huisman pattern as their Figure 5, “Bayes factors for a small true effect size” (reproduced here as Fig. 1) and discuss the results as follows:

“For small to moderate sample sizes, the Bayes factor supports the null. As the sample size becomes exceedingly large, however, the small deviations from the null are consequential, and the Bayes factor yields less support for the null. In the large-sample limit, the Bayes factor favors the alternative, since the null is not exactly true. *This behavior strikes us as ideal*. With smaller sample sizes that are insufficient to differentiate between approximate and exact invariances, the Bayes factor allows researchers to gain evidence for the null. This evidence may be interpreted as support for at least an approximate invariance. In very large samples, however, the Bayes factor allows for the discovery of small perturbations that negate the existence of an exact invariance. In sum, the Bayes factor favors the more parsimonious null-model description with small observed effect sizes unless the sample size is so large that even these small effects are not compatible with the null relative to the alternative.” (Rouder et al., 2009, p. 233, italics added for emphasis)

## Concluding comments

Huisman (2022) postulated that the expected evidence in favor of a true alternative hypothesis ought to increase monotonically with sample size. This intuition is at odds with probability theory, leaving us with two possible resolutions. Huisman chooses the first and concludes that the Bayes factor –which was expressly developed to be a valid measure of evidence– is an *invalid* measure of evidence. We suggest the second resolution, which is to accept that probability theory can be counterintuitive; researchers cannot know with certainty what is hidden in their data until they have conducted all the required calculations. As discussed throughout the statistical literature over the past 80 years, the increasing support in favor of the incorrect hypothesis comes about not because of the Bayes factor, but because the alternative hypothesis specifies a prior distribution that is relatively wide. Paying the price of vagueness is a Bayesian manifestation of Ockham’s razor; without it, researchers would not be able to quantify evidence in favor of a true null hypothesis.

### Disclosures

## References

[CR1] Etz, A., & Wagenmakers, E. J. (2017). J. B. S. Haldane’s contribution to the Bayes factor hypothesis test. *Statistical Science,**32*, 313–329.

[CR2] Evans M (2015). Measuring statistical evidence using relative belief.

[CR3] Holcombe, A. O., Kovacs, M., Aust, F., & Aczel, B. (2020). Documenting contributions to scholarly articles using credit and tenzing. *PLoS One,**15*, 10.1371/journal.pone.0244611 PMC7775117 33383578

[CR4] Huisman, L. (2022). Are $$p$$-values and bayes factors valid measures of evidential strength? *Psychonomic Bulletin & Review*, 1–10. 10.3758/s13423-022-02205-x 36417167

[CR5] Jeffreys, H. (1939). Theory of probability. *Oxford University Press*, Oxford, UK, 1 edition.

[CR6] Jeffreys, H. (1948). Theory of probability. *Oxford University Press*, Oxford, UK, 2 edition.

[CR7] Jeffreys, H. (1961). Theory of probability. *Oxford University Press*, Oxford, UK, 3 edition.

[CR8] Kass RE, Raftery AE (1995). Bayes factors. Journal of the American Statistical Association.

[CR9] Ly A, Verhagen J, Wagenmakers EJ (2016). Harold Jeffrey’s default Bayes factor hypothesis tests: Explanation, extension, and application in psychology. Journal of Mathematical Psychology.

[CR10] Morey, R. (2015). All about that “bias, bias, bias” (it’s no trouble). http://bayesfactor.blogspot.com/2015/04/all-about-that-bias-bias-bias-its-no.html

[CR11] Myung IJ, Pitt MA (1997). Applying occam’s razor in modeling cognition: a bayesian approach. Psychonomic Bulletin & Review.

[CR12] Rouder JN, Speckman PL, Sun D, Morey RD, Iverson G (2009). Bayesian $$t$$ tests for accepting and rejecting the null hypothesis. Psychonomic Bulletin & Review.

[CR13] van Ravenzwaaij, D., & Wagenmakers, E. J. (2022). Advantages masquerading as “issues” in Bayesian hypothesis testing: A commentary on tendeiro and kiers (2019). *Psychological Methods,**27*, 451–465. 10.1037/met0000415 34881956

[CR14] Vandekerckhove, J., Rouder, J. N., & Kruschke, J. K. (2018). Editorial: Bayesian methods for advancing psychological science. *Psychonomic Bulletin & Review,**25*, 1–4. 10.3758/s13423-018-1443-8 29450790

[CR15] van Dongen, N., Sprenger, J., & Wagenmakers, E.J. (2022). A Bayesian perspective on severity: Risky predictions and specific hypotheses. *Psychonomic Bulletin & Review*, 1–18. 10.3758/s13423-022-02069-1 PMC10104935 35969359

[CR16] Wagenmakers EJ, Lodewyckx T, Kuriyal H, Grasman R (2010). Bayesian hypothesis testing for psychologists: A tutorial on the Savage-Dickey method. Cognitive Psychology.

[CR17] Wagenmakers, E.J. & Ly, A. (2022). History and nature of the Jeffreys–Lindley paradox. *Archive for History of Exact Sciences*, pages 1–48. 10.1007/s00407-022-00298-3 PMC11442617 39355509

[CR18] Wagenmakers EJ, Marsman M, Jamil T, Ly A, Verhagen J, Love J, Selker R, Gronau Q, Smira M, Epskamp S, Matzke D, Rouder J, Morey R (2018). Bayesian inference for psychology. Part I: Theoretical advantages and practical ramifications. Psychonomic Bulletin & Review.

[CR19] Wagenmakers EJ, Morey RD, Lee MD (2016). Bayesian benefits for the pragmatic researcher. Current Directions in Psychological Science.

